# “Life continues”: Patient, health care and community care workers perspectives on self-administered treatment for rifampicin-resistant tuberculosis in Khayelitsha, South Africa

**DOI:** 10.1371/journal.pone.0203888

**Published:** 2018-09-14

**Authors:** Erika Mohr, Leigh Snyman, Zodwa Mbakaz, Judy Caldwell, Virginia DeAzevedo, Yulene Kock, Laura Trivino Duran, Emilie Venables

**Affiliations:** 1 Médecins Sans Frontières (MSF), Khayelitsha, South Africa; 2 City of Cape Town Health Department, Cape Town, South Africa; 3 Provincial Government of the Western Cape Department of Health, Cape Town, South Africa; 4 Southern Africa Medical Unit, Médecins Sans Frontières (MSF), Cape Town, South Africa; 5 University of Cape Town (UCT), Division of Social and Behavioural Sciences, School of Public Health and Family Medicine, Cape Town, South Africa; International Union Against Tuberculosis and Lung Disease, INDIA

## Abstract

**Background:**

Self-administered treatment (SAT), a differentiated model of care for rifampicin-resistant tuberculosis (RR-TB), might address adherence challenges faced by patients and health care systems. This study explored patient, health-care worker (HCW) and community care worker (CCW) perspectives on a SAT pilot programme in South Africa, in which patients were given medication to take at home with the optional support of a CCW.

**Methods:**

We conducted a mixed-methods study from July 2016-June 2017. The quantitative component included semi-structured questionnaires with patients, HCWs and CCWs; the qualitative component involved in-depth interviews with patients enrolled in the pilot programme. Interviews were conducted in isiXhosa, translated, transcribed and manually coded.

**Results:**

Overall, 27 patients, 12 HCWs and 44 CCWs were enrolled in the quantitative component; nine patients were also interviewed. Of the 27 patients who completed semi-structured questionnaires, 22 were HIV-infected and 17 received a monthly supply of RR TB treatment. Most HCWs and CCWs (10 and 32, respectively) understood the pilot programme; approximately half (n = 14) of the patients could not correctly describe the pilot programme. Overall, 11 and 41 HCWs and CCWs reported that the pilot programme promoted treatment adherence. Additionally, 11 HCWs reported that the pilot programme relieved pressure on the clinic. Key qualitative findings highlighted the importance of a support person and how the flexibility of SAT enabled integration of treatment into their daily routines and reduced time spent in clinics. The pilot programme was also perceived to allow patients more autonomy and made it easier for them to manage side-effects.

**Conclusion:**

The SAT pilot programme was acceptable from the perspective of patients, HCWs and CCWs and should be considered as a differentiated model of care for RR-TB, particularly in settings with high burdens of HIV, in order to ease management of treatment for patients and health-care providers.

## Introduction

Rifampicin-resistant tuberculosis (RR-TB) until recently, required two-years of treatment, often resulting in debilitating side effects [1–3]. Additionally, patients are required to attend a clinic on a daily basis to receive treatment under directly observed therapy (DOT) [4]. These factors can negatively impact treatment adherence [5], resulting in loss to follow-up (LTFU) [6]. Other factors associated with LTFU are gender, age, previous TB episodes, substance abuse [7], and distance from the clinic [8]. Additionally, costs incurred by the patient during RR-TB treatment and care, including transportation costs [9], impact LTFU as there is often no social compensation for those expenses [10].

The reasons for LTFU are multi-factorial and attributable to individual and health system challenges [2,6]. In South Africa, a setting with a high burden of HIV, approximately 30% of RR-TB patients experience a treatment outcome of LTFU [11–13]. Two thirds of patients LTFU in Khayelitsha, South Africa, successfully endured and completed the painful six month injectable phase of treatment and only interrupted treatment once they reached the continuation phase [14]. The clinical status of most patients generally improves after a few months on effective treatment thus they are able to carry out normal activities of daily living by the time they reach the continuation phase. Anecdotally, most patients feel ready to resume their daily routines and responsibilities (i.e. working, studying, and caring for and supporting their families), long before the completion of their treatment course. However, these patients are still required to receive their oral medications in a clinic every single day under DOT, which becomes increasingly difficult to do over an extended period of 24 months [15]. DOT is the treatment strategy recommended by the World Health Organization (WHO) for RR-TB globally [16] and in the Strategic Framework for the management of RR-TB in South Africa[15]; however there is conflicting information about the utility of DOT in various programmatic settings [2,17,18]. Implementing differentiated models of care by intensifying support for RR-TB patients and offering patient centered care should be considered in RR-TB treatment programmes, as is common in HIV programmes[19– 22].

A pilot programme to integrate adherence support for RR-TB patient into the existing TB/HIV integrated adherence framework was undertaken in 2012 by the international medical humanitarian aid organisation *Médecins Sans Frontières* (MSF). The overall goal of this self-administered treatment (SAT) pilot programme, a differentiated model of care for RR-TB, was to encourage RR-TB patients to take treatment without having to attend the clinic daily. RR-TB counselors provide adherence counseling at the end of the completion of the intensive phase, at which time a patient is assessed for placement out of DOT. Patients were considered eligible for SAT if they were clinically stable (confirmed culture negativity and clinically improving), had few adverse events associated with treatment and if they had a favourable adherence record for RR-TB and concomitant diseases, as subjectively determined by the treating physician. If accepted for the SAT pilot programme, MSF RR-TB counselors conduct adherence counseling with the patients, stressing the importance of treatment and providing detailed information regarding the programme. Patient pill-boxes were allocated to ease the management of the RR-TB treatment supply at home. Patients in the programme were given the option of being supported by community care workers (CCWs). If a patient decides that they want CCW support, the CCW will visit them in their home once accepted into the programme to review the patient’s adherence. This process has been described in detail in a previous publication[17]. It was anticipated that this model of care would relieve some of the burden that receiving daily DOT places on the clinic staff, as well as on the patients. Previous publications and reports emerging from the programme showed that patients who received SAT did not experience increased rates of LTFU when compared to patients that received DOT [17,23].

To date there have been very few studies which have explored the experiences of SAT from the perspective of beneficiaries and those implementing the pilot programme. The aim of this study was to understand the experiences of those involved in the SAT pilot, including challenges and benefits, from the perspectives of patients, health care workers (HCWs) and CCWs.

## Materials and methods

### Design

This was a concurrent mixed methods study [24] conducted between July 2016 and June 2017 to describe patient, HCW and CCW perspectives of the SAT pilot programme in Khayelitsha, South Africa. The quantitative component of the study included semi-structured questionnaires, including open-ended questions, with patients, HCWs and CCWs and the qualitative component involved in-depth interviews (IDIs) with patients. The rationale for mixed methods was to be able to explore issues included in the semi-structured questionnaires in more detail with patients, so as to gain a better understanding of their experiences of the programme.

### Setting

Khayelitsha is a peri-urban township located on the outskirts of Cape Town, South Africa with a population of approximately 450,000 people, most of whom reside in informal settlements [25]. Approximately 200 patients are diagnosed with RR-TB annually, with a case notification rate of 55/100,000 [12]; HIV prevalence is 70% among those treated for RR-TB [13]. Patients are treated for RR-TB in 11 different primary health care facilities in Khayelitsha, as described in previous publications [12]. RR-TB counselors provide treatment literacy and adherence support at key points during patients’ treatment journeys [26].

### Programme description

Only a sub-set of all the patients treated for RR-TB in Khayelitsha were deemed eligible for SAT based on their adherence record, clinical status, and frequency of adverse events that might require ongoing monitoring. The selection criteria for inclusion in the programme were inherently biased as only adherent patients were considered in order to determine is this model of care would be feasible for use among uncomplicated RR-T cases. Further details regarding this progamme have been detailed in previous publications [17][23].

### Semi-structured questionnaires and interview guides

The semi-structured questionnaires and in-depth interview guides were developed by members of the study team and reviewed with the RR-TB counselors for content (**S1 and S2 Files**). The semi-structured questionnaires and the IDI guides were translated into isiXhosa (the local language) and back-translated into English to ensure accuracy. The in-depth interview guide was also piloted by the research assistant to ensure the questions were understandable and followed a logical flow. The semi-structured questionnaires and in-depth interviews focused on similar themes. These included programme details, past and current adherence challenges, the roles of the HCWs and CCWs in the programme, and experiences and challenges with the programme in order to highlight its strengths and weaknesses. The IDIs then addressed similar themes in more detail with the patients.

### Participant selection

Patients were selected for recruitment to the study if they had been enrolled in the SAT pilot programme for a minimum of six months, had not been discharged from RR-TB treatment (were still in care from July 2016-June 2017) and were 18 years of age or older. Convenience sampling was used to select a sub-set of patients who had completed the semi-structured questionnaires for IDIs.

HCWs and CCWs were selected for participation in semi-structured questionnaires if they had been employed at the health care facility for at least six months and had experience working in the clinic after the implementation of SAT from July 2016-June 2017.The HCWs enrolled included doctors and RR-TB nurses; however, their position was not specified on the semi-structured questionnaires.

### Recruitment

Eligible patients, HCWs and CCWs were identified by a member of the study team through routine patient records and with the assistance of the programme staff. An independent, female research assistant with training in qualitative methods contacted and recruited those who were interested. She was not involved in routine RR-TB patient care in Khayelitsha and was fluent in isiXhosa and English. Potential participants were not familiar with the research assistant prior to the research, and during recruitment she explained that she was involved with the study but not the provision of care, thus limiting any presumptions of bias. Potential participants were contacted telephonically.

There were challenges in recruiting eligible patients for the study. In total we identified 55 patients for enrolment, but 28 patients were not enrolled due to the following reasons: patients were discharged before they could be interviewed (n = 13), they could not be contacted with the telephone numbers provided (n = 11), they had travelled out of the study area (n = 3) or they repeatedly cancelled appointment dates (n = 1). Additionally, between November 2016 and February 2017 there were very few patients enrolled in the SAT pilot programme due to seasonal mobility within South Africa at that time of the year, therefore there were few eligible patients enrolled in SAT meeting the eligibility criteria for inclusion in this study. The independent research assistant implemented the semi-structured questionnaires and conducted the interviews.

### Interviews and data collection

All HCW and CCW questionnaires were self-administered and completed manually. The IDIs were conducted in the patient’s home or in the clinic, depending on the patient’s preference. IDIs were audio-recorded and were stopped once saturation was reached. The IDIs and questionnaires that were conducted in isiXhosa were later translated into English and reviewed by the research team to identify and correct any discrepancies in translation. Handwritten notes from the research assistant were also consulted after each interview, but not transcribed. A ‘two-step’ transcription and translation process took place, in which interviews were transcribed in isiXhosa before being translated into English. Each interviewee was only interviewed once and interviews lasted a median of 55.5 minutes. Transcripts were reviewed within the research team, but were not shared with the participants for further validation afterwards. No other people apart from the research assistant and the interviewee were present during the IDIs.

### Data analysis

Quantitative data were analysed using STATA version 14.1. Continuous data were presented as medians and interquartile range while categorical data were presented as frequencies and proportions. Qualitative data were manually coded by two investigators and a thematic approach to analysis was utilized; basic themes emerging from the data were grouped into organizing themes and then into a global overarching theme as described by Attride-Stirling [27]. This study was conducted in line with the COREQ guidelines for the reporting of qualitative data [28].

### Ethics

This study was approved by the MSF Ethical Review Board (#1607) and the Human Research Committee of the University of Cape Town (922/2015). Written informed consent was obtained from all study participants before data collection began.

## Results

We present quantitative and qualitative results below: quantitative data are taken from the semi-structured questionnaires, and qualitative data from the in-depth interviews and open-ended questions from the questionnaires.

### Clinical and demographic characteristics

A total of 27 patients, 12 HCWs, and 44 CCWs were included in the study (**Tables 1 & 2**). Of note, 22 patients were HIV-infected. The median time from RR-TB treatment initiation to enrolment into the SAT pilot programme was 7.4 (5.7–10.8) months and 17 patients in the SAT pilot programme had received a monthly supply of RR-TB medication. Of the HCWs participating, 10 were female and the median length of time in their current position was two years. The CCWs had a median of six years working in the community, and 41 CCWs were female.

**Table 1:** Clinical and demographic characteristics of enrolled patients.

Characteristics	Number of participants
***RR-TB patients***	***n = 27***
**Sex**	
Male	15
Female	12
**Median age (years) at interview (IQR)**	38
**RR-TB disease classification**	
Xpert unconfirmed	1
Rifampicin-mono resistant tuberculosis	4
Multi-drug resistant tuberculosis	17
Pre-extensively drug-resistant tuberculosis (fluoroquinolone)	2
Pre extensively drug-resistant tuberculosis (injectable)	2
Extensively drug-resistant tuberculosis	1
**Previous RR-TB treatment history**	
None	15
Previously treated with first line anti-tuberculosis drugs	8
Previously treated with second line anti-tuberculosis drugs	4
**HIV Status**	
Positive	22
Negative	5
**On ART at RR-TB treatment initiation**	22
**Median months from RR-TB treatment to SAT (IQR)**	7.4 (5.7–10.8)
**Supply of RR-TB medications**	
Weekly	9
Fortnightly	1
Monthly	17
**Supported by a Community Care Worker (Yes)**	22
***Health Care Workers***	***N = 12***
**Sex**	
Male	2
Female	10
**Median age (years) at time of interview (IQR)**	36.5 (30.5–45.5)
**Median years employed in the health care facility**	2 (1.2–3.5)
***Community Care Workers***	***N = 44***
**Sex**	
Male	3
Female	41
**Median age (years) at interview (IQR)**	44 (35.5–51)
**Median years employed working in the community**	6 (4–10)

Abbreviations: RR-TB, rifampicin-resistant tuberculosis; IQR, interquartile range; ART, anti-retroviral therapy; SAT, self-administered treatment.

**Table 2:** Characteristics of the enrolled patients who completed the in-depth interviews.

Patient Number	Gender	Age
1	Female	33
2	Male	34
3	Male	44
4	Male	43
5	Female	60
6	Male	31
7	Female	45
8	Female	43
9	Male	44

In addition, a total of four women and five men from the quantitative sample took part in in-depth interviews (**Table 2**). The median age of these interviewees was 42.

### Quantitative results

#### Knowledge and understanding of the SAT pilot programme

Questionnaire data showed that 13 patients knew that the SAT pilot programme was a new programme being initiated in some clinics in Khayelitsha; 20 stated that they understood why they were given a supply of medication to take at home. Key reasons for inclusion as self-identified by patients in open-ended questions included: the belief that they were selected by HCWs due to their good adherence (n = 5), they personally requested SAT (n = 4), improved health (n = 4) and logistical work or travel challenges hindering them from attending the clinic (n = 4). Only two and three patients responded that they were offered SAT because they completed the first six months of treatment or because a HCW had identified them as eligible for the pilot, respectively.

Overall, 10 HCWs and 32 CCWs were able to identify at least one of the eligibility criteria for enrollment in SAT, the most common being the completion of the injectable phase or at least the first six months of RR-TB treatment (open-ended questions). Two HCWs and one CCW reported that patients were eligible for SAT after two weeks of treatment; another CCW reported that patients were eligible for SAT three days after RR-TB treatment initiation.

#### Support for patients in the SAT pilot programme

Patients reported that they found it helpful for the RR-TB counselor to explain their RR-TB treatment (n = 26) and RR-TB pillbox (n = 23), and how to remember to take treatment once in the SAT pilot programme (n = 25). Only one patient reported that they had never received the support of a counselor and three patients reported that they not received a pillbox. In addition, 26 patients reported that they received treatment support from someone outside of the programme.

Of the 22 patients who received the support of a CCW (Table 1), 15, 13, and 10 reported that the CCWs ‘sometimes’ visited them weekly/monthly for their scheduled visits, checked their patient card and checked their pill box, respectively. Conversely, the majority of CCWs (n = 30) reported that they do not struggle to see their patients at home. Only seven patients reported that the CCW ‘always’ visited them weekly/monthly. Twenty patients responded to open-ended questions saying that they felt that the CCW supported them by checking to see if they had taken their tablets, encouraging them, being open to have lengthy conversations with them and by being available at weekends.

#### Benefits of SAT

All of the study participants said that they liked getting a supply of RR-TB medications to take at home. Additionally, 23 reported that they preferred to take their treatment at home every day, while three and one respectively said they were ‘not sure’ and or would rather take their treatment in the clinic. Patients listed the following reasons in an open-ended question for not wanting to take treatment in the clinic: queues and congestion; time constraints and fatigue from traveling to and waiting in the clinic; discontent when nurses watched them taking treatment; inability to work and lack of flexibility.

In total, 25 patients reported that SAT made it easier for them to take their treatment. Patients described that it was easier to take treatment in the comfort of their own homes because there was flexibility, they could return to their daily activities easily and if they were sick they could manage their common side-effects such as nausea and vomiting at home. One patient who wanted to take treatment in the clinic stated in an open-ended question that it would not be appropriate to take treatment at work.

Open-ended responses to questionnaire data revealed that all HCWs and CCWs (n = 12 and n = 44) found the SAT pilot programme useful for RR-TB patients, because it made it easier for them to take their treatment and allowed them greater independence and autonomy during their treatment. Additionally, they reported that patients were required to spend less time in the clinic and could return to their normal activities, including regular employment. The HCWs self-identified the following benefits of the programme in their responses to an open-ended question: decongestion in the clinic (n = 10), decreased LTFU and better patient compliance to medication (n = 10), decreased work load (n = 4), improved patient satisfaction (n = 2) and the ability to spend more time with sick patients (n = 2).

In addition, CCWs self-identified the following benefits in an open-ended question: improved personal knowledge (n = 32), the ability to act as a care provider (n = 28), being seen as ‘important’ and building relationships and trust with patients (n = 12), seeing patients taking their treatment and thus reducing LTFU (n = 8) and seeing patients’ health improve (n = 8).

#### Adherence challenges

Adherence remained a challenge for some of the patients in the pilot programme, with 15 patients indicating that they sometimes forgot to take treatment, or that they remembered but were unable to take it. Reasons for not always taking their RR-TB treatment included travel, work, feeling sick as a result of the treatment and forgetting to take it. The main challenges that the CCWs identified in open-ended questions for patients enrolled in the SAT pilot programme in taking their treatment were: side-effects (n = 23), lack of food at home/hunger (n = 22), lack of support/rejection by family (n = 18), substance use (n = 16) and poverty/a lack of work (n = 9).

Overall, 11 HCWs and 41 CCWs reported that the SAT pilot programme assisted RR-TB patients in adhering to their treatment.

#### Eligibility criteria

When asked about eligibility criteria, five HCWs thought that the eligibility criteria into the programme should be more flexible and allow for a more individualised management of patients, particularly for those on injectable free regimens. Conversely, 38 CCWs thought that the eligibility criteria should remain as they were. In addition to the data presented above, key reasons identified in open-ended questions included the feeling that patients should first become familiar with their treatment before being enrolled into the SAT pilot programme and to ensure that they were adherent and no longer sick.

When asked if they thought that the SAT pilot programme should be rolled-out and made available to other RR-TB patients, 19 patients responded negatively. The majority of those patients (n = 14) identified concerns about the adherence of others as the main reason that they should not receive SAT.

### Qualitative results

We now explore the qualitative findings, using both data from in-depth interviews with patients and open-ended questionnaire responses from patients, HCWs and CCWs. The main themes presented are understanding of the programme and its perceived benefits; managing side-effects; peer support; motivation and empowerment and programmatic considerations.

#### Understanding of the pilot programme

This patient reflected on his understanding of the pilot programme during an IDI:

You are given treatment to take at home because they saw that you are able to stand on your own [autonomous] …They [nurses] will monitor that you will come on the refill date to take more. (P1)

When HCWs and CCWs were asked to describe how SAT differs from the standard of care during the open-ended questions in the semi-structured questionnaire, responses included: taking treatment at home after six months of clinic treatment, taking care of one’s self, not having to come to the clinic as often, improved patient education regarding treatment, self-administration of treatment rather than DOT and CCW support. One CCW reported that the ‘*difference is that he is eating at home*. *He is taking care of himself*’.

#### Perceived benefits of the pilot programme

Patients, HCWs and CCWs discussed what they considered to be the benefits of the programme in the open ended questions. Patients began by describing their experiences of DOT before the SAT pilot as a ‘*struggle*’, saying that they ‘*suffered*’ as a result of the treatment. One interviewee (P3) said that before she was enrolled in SAT ‘*it was not easy’*. Patients described going to the clinic as ‘*restrictive*’ in terms of their daily routines, especially if they were working.

Additionally, the IDIs revealed that patients enjoyed being part of the SAT pilot programme, describing their involvement with words such as ‘*happy*’, ‘*relieved’*, ‘*positive*’, ‘*motivating*’ and ‘*encouraging*’. Only one patient did not like the pilot programme, as he found it more restrictive than collecting and taking his treatment at the clinic and still considered SAT to be a form of *‘discipline’*. Patients enjoyed the autonomy and freedom that taking their medication at home offered them, and appreciated being able to set their own schedule for treatment.

This patient (P1) described the effect SAT had upon her daily routine:

I was lazy when I woke up in the morning and thought about going to the clinic. But when they are here at home in the wardrobe, I take my medication.

A second patient (P5) also explained how the programme benefitted her:

It helped me in the state I was in. I was not a person [felt dehumanised]. Now I can’t stop taking my treatment.

#### Management of side-effects

Patients found it was easier to manage the side-effects of RR-TB treatment when they were able to take their medication at home, as they could eat directly after taking their treatment. Being at home instead of in the clinic also gave these two patients the freedom to eat when they liked:

I prefer to take my treatment at home because of nausea…I can drink juice sometimes (P4)

I prefer to eat first and then take my medication. I vomit them if I don’t eat (P5)

One female patient (P8) also highlighted the challenge of managing her treatment and the need to eat at the same time:

Previously it was very difficult when I came to take my treatment there at the clinic… Sometimes I came without having time to eat, because I was rushing to the clinic.

Another patient (P8) referred to her discontent regarding the amount of pills she needed to take every day:

These pills are many. I do not want to eat them all at the same time as they make me vomit. I need to take them portion by portion.

#### Peer support

Patients appreciated the help provided to them by the RR-TB counselor, stating that the milestone counseling session in which they were reviewed for SAT enrollment re-motivated, encouraged and educated them.

When asked what role they play in supporting RR-TB patients enrolled in SAT, HCWs self-identified in open ended questions that they are involved in the clinical oversight, monitoring, review and prescribing for patients. One HCW stated that their role was ‘t*o ensure a high standard of care is maintained and that a good clinical outcome is achieved*’ while another HCW reported that ‘w*orking with counselors*, *we allay the fears of dependency syndrome [disability grant] when we allow patients to self-take treatment*’.

Interviews revealed that support for patients came from people including mothers, wives, sons, partners and friends, as this male patient describes:

My wife supports me by always reminding me of the time to take my pills and saying that I must ‘hang in there’. (P3)

One patient replied on the semi-structured questionnaire: ‘*I like it [SAT] because it doesn't make any changes in our daily routine*. *Life continues*. *It made things easier*’.

#### Motivation and empowerment

Interviewees discussed how the SAT pilot programme affected their own motivation and adherence, and HCWs and CCWs reflected upon the effect on their patients. Whilst one individual felt that they were being ‘*forced*’ to take their treatment through being involved in the SAT pilot programme, all other interviewees had a positive, empowering experience with the programme.

As highlighted above, patients reported that belonging to the SAT pilot programme gave them freedom and flexibility to take their treatment on their own, in their own time and allowed them to return to their daily activities.

Patients also reported similar feelings when completing the open-ended questions, mentioning previously feeling ‘*pressured’* to take their treatment at the clinic. One patient believed that SAT motivated them to take their treatment, stating that they *‘love it because I do not forget*, *I motivate myself*. *When the time comes to take my pills I take them*’.

Interestingly, one patient did not like going to the clinic because it was stressful: ‘*What I like [about SAT] is that I do not get stressed meeting people who look like they are not getting cured*. *It seems like that can take me back*, *I am scared of it*.*’*

Generally, there was a perception amongst HCWs and CCWs that the programme was leading to a reduction in LTFU and an increase in the cure rate, which was rewarding for staff involved in its implementation.

#### Programmatic considerations

In the open-ended questions, several CCWs expressed their concerns regarding the SAT pilot programme being stopped and the implications this would have on the patients and the community, with one CCW stating that ‘*my concerns are if it can be stopped many people can lose their lives*, *or infect many people in the community*’.

In general, HCWs and CCWs believed that the SAT pilot was successful. One HCW reported that ‘*[we] need flexibility depending on the condition of patient*, *family support*, *reliability; the use of bedaquiline and delamanid needs consideration*’ for inclusion in SAT.

When asked about the role that they play in supporting patients, key themes emerged in the CCWs responses to open-ended questions including: visiting patients at home, supporting and encouraging them, counting their pills, ensuring patients are taking their medications, helping people to be cured and preventing them from being LTFU.

## Discussion

Our mixed-methods study describes the experiences of patients, HCWs and CCWs involved in self-administered RR-TB treatment in a peri-urban township with high rates of HIV co-infection in South Africa. The data suggest that the programme was acceptable and beneficial to patients because it gave them more autonomy over their treatment, allowed them to return to their daily activities and enabled them to better manage their side-effects. The programme was also believed, by HCWs and CCWs, to reduce congestion in the clinic and reduce the burden of clinic visits on patients.

The burden of RR-TB treatment on patients has been well-documented in South Africa and elsewhere. Living with dual diseases further adds to the load carried by patients [29]; 82% of those included in this study were HIV-infected. In this study, patients described DOT as restrictive and limiting daily activities of life. These challenges were reduced and in some cases relieved by enrolment into the SAT pilot programme. A previous study which investigated whether patients preferred home or hospital based care showed the acceptability of and preference towards home-based care [8]. Literature reflects on the complexities DOTs brings to patients’ lives [2,5] but it lacks discussion regarding the impact of SAT on the lives of those patients’. To our knowledge this is the first study to investigate health care providers and patients perspectives of a SAT pilot programme.

The majority of participants expressed that SAT motivated patients to have autonomy over their treatment journeys. The CCWs also felt motivated and empowered through their provision of care to these patients in need. The majority of HCWs and CCWs shared a similar, unfounded perception that LTFU rates were declining and cure rates were increasing among the patients in the SAT pilot programme; however this is not backed up by previously published quantitative data [17]. This presumption alone, given the high rates of LTFU and mortality with concurrent high rates of HIV co-infection, may have motivated these care providers in the provision of support to patients.

Implementing pilot programmes such as SAT require thorough training of staff, including refresher trainings, as well as clear explanations to patients. This is to ensure that implementers and beneficiaries alike understand the purpose of the pilot programme and the eligibility criteria [30]. Whilst our data show that almost all patients were appreciative of SAT, they were not always sure why they had been enrolled. This could point to a gap in the quality of the counseling provided with the SAT pilot programme or a lack of clarity during the recruitment phase. The lack of a clear understanding regarding the eligibility criteria among HCWs and CCWs reflects the need for enhanced training before implementation and enhanced follow-up afterwards. Studies conducted in South Africa show that task shifting patient support activities to community workers is an essential component of differentiated HIV/TB care in primary health care settings [21]. However, task shifting requires training packages that include supervision, mentoring and support [31–34]. This need is clearly reflected by several patients who reported that they did not see their CCW regularly, suggesting that more clarity about the CCWs role and relationship with patients is needed to ensure sufficient follow-up. It is important that CCWs receive frequent training on the provision of patient support, and that they themselves stay motivated and function as a team in order to stay engaged in their work. Finally, a study conducted in the Western Cape showed that combining quality improvement programmes alongside pilot programmes ensures improved outcomes and could be reflective of the limitation in the Khayelitsha setting [30].

Differential service delivery is becoming increasingly common in many settings with large HIV and TB burdens, but has not been widely implemented in RR-TB programmes. Differentiated models of care, many of which have been piloted in Khayelitsha and in other settings in Southern Africa, include clinic- and community-based clubs and Community ART Groups (CAGs) [19,20,35,36]. This study provides policy makers and programme implementers with information on a model of care which could be included in a differentiated model of care package for RR-TB. Such models should be considered in settings in which there are high burdens of RR-TB and limited public health resources. Whilst we recognise that our results are from an urban setting where it is easier to access patients at home than it may be in remote, rural areas, we believe this level of task-shifting can happen in other settings too. Our study showed that most but not all patients believed that SAT was beneficial, thus it is important to ensure that patients are involved in discussions about which model of care is best suited to them. Interestingly, whilst patients appreciated the programme, they did not think it should be expanded to everyone, again highlighting the importance of having clear recruitment and eligibility criteria and processes. This could also suggest a sense of pride and ownership over the programme, with current SAT beneficiaries not wanting the programme to be rolled out to others.

The data highlighted the significance of having someone, who was usually a relative, to support the patient with taking RR-TB treatment at home. This support person typically offered psycho-social support as well as daily reminders to take treatment, or provided help with preparing meals after taking treatment to help ease side-effects. The key role of support people or ‘treatment partners’ as they are often referred to in HIV programmes, has also been explored elsewhere [37]. The importance placed on these support networks suggests that they are essential and need to be considered when implementing similar programmes in other contexts.

The majority of the participants expressed that they were satisfied with this pilot programme and that it should be continued with little modification made to the design. Some patients reported that they sometimes forgot or were unable to take their treatment, and still struggled at times to manage their side-effects, showing that adherence remained challenging for some. This phenomena is also reflected in the data for other long term therapies [38].

These findings highlight the need for continued adherence support for patients once they are enrolled onto SAT, and the need to address the reasons for continued treatment interruption [39]. The CCWs highlighted some well-documented challenges faced by patients in the programme [1,29]. These challenges did not speak to the implementation of the programme, however, but to challenges faced by RR-TB patients in general. Whilst this study has shown that it is both feasible and acceptable for RR-TB patients to self-administer their RR-TB treatment at home instead of attending a clinic, there is still the need for improved, shorter and injectable-free RR-TB regimens with fewer side effects, fewer drugs and improved efficacy [40]. This differentiated model of care could also be adapted and offered as an option to patients receiving the short course regimen which has now been rolled out in the Western Cape and globally. As our data showed, many of the ongoing challenges faced by patients, such as side-effects and pill burden could not be resolved through the provision of SAT alone. Another point which was mentioned in this evaluation by CCWs, but not discussed at length in this paper, was the effect of substance use upon patients’ treatment adherence. There is a need for programmes which specifically focus on the provision of support to patients with substance use disorder (SUD) in order to address the multi-faceted adherence challenges SUD poses, as has also been documented in HIV cohorts [41].

There are several strengths and weaknesses to this study. As we only included patients who were still enrolled in the SAT pilot programme, and currently employed HCWs and CCWs, one of the strengths is that we did not have challenges with recall bias. Although we had a small sample size, we feel that the patients enrolled were representative of the RR-TB patients enrolled in SAT, as there were few eligible patients due to the small numbers of patients enrolled in the programme. Additionally, we were able to triangulate the data from the different groups. One limitation was a potential bias in the selection and recruitment of participants. The overall SAT pilot programme excluded patients with adherence challenges, who would be considered the most vulnerable patients, therefore the findings from this study and pilot programme cannot be generalised to all patients with RR-TB. Future studies should investigate programmes which include patients that might benefit more from this pilot programme, such as those with poor adherence. Additionally, those who participated in this study might have been more likely to discuss the pilot programme favorably and might have responded with answers they think the interviewer wanted to hear, even though efforts were taken during the consent process to ensure that this did not happen.

## Conclusions

Findings from this mixed-methods study showed that the SAT pilot programme, a differentiated model of care for the treatment of RR-TB post completion of the injectable phase of treatment, was considered to be beneficial from the perspectives of patients, HCWs and CCWs. These findings should be interpreted with caution as the SAT pilot programme only enrolled adherent patients and thus the views represented here are not from patients who had adherence challenges, although such patients may benefit more from such a programme in future. Overall, patients expressed that they felt motivated and that it was easier to take their treatment and manage their side effects at home. Additionally, HCWs indicated that SAT alleviated pressure placed on them and the clinic allowing them more time to spend with sick patients. Finally CCWs felt empowered and motivated when providing support to patients enrolled in the programme, and highlighted that their involvement led to substantial knowledge gain. This differentiated model should be considered in other settings with high burdens of RR-TB and HIV and implementation should be coupled with thorough sensitisation, training, and mentoring to ensure that patients and implementers understand the purpose of the programme and who is eligible for participation. The ongoing challenges associated with RR-TB treatment, including adherence and side-effects, highlight the need for new and improved regimens with a shorter treatment duration. Future studies will be needed to determine the feasibility and acceptability of this model in other settings with RR-TB.

## Supporting information

S1 File Standardized satisfaction questionnaires for patients, HCWs, and CCWs in English and isiXhosa. (DOCX)

S2 File In-depth interview guide for patients in English and isiXhosa. (DOCX)

S3 File Quantitative data file. (XLSX)
