# Supplementary material for: “Life continues”: Patient, health care and community care workers perspectives on self-administered treatment for rifampicin-resistant tuberculosis in Khayelitsha, South Africa
Source: PLoS One. 2018 Sep 14;13(9):e0203888. doi: 10.1371/journal.pone.0203888 (PMC6138394; doi:10.1371/journal.pone.0203888)
Supplement: S1 File — (DOCX) [file pone.0203888.s001.docx]

**Annex 1: Patient, Health Care Worker and Community Care Worker Satisfaction Questionnaires**

**Patient Satisfaction Questionnaire (English)**

**Rifampicin-Resistant Tuberculosis (RR-TB) Self-Administered Treatment (SAT) Pilot Programme**

**Rifampicin Resistant Tuberculosis (RR-TB) Self-Administered Treatment Pilot Programme**

Clinic: ___________________

Patient Identification Number: ______________________________

Age: ____________________________ Sex: Male _______ Female _______________

Date: ___________________

**Section 1: Initiation into Self-Administered Treatment (SAT) Programme**

**The following questions are general questions about the Self-Administered Treatment (SAT) Pilot Programme**

1. Are you getting a **weekly** or a **monthly** supply of RR-TB treatment from your clinic to take at home?
   1. Weekly
   2. Monthly
   3. Other: _____________________________________________________________

**Prompt:** Giving people a supply of RR-TB treatment to take at home is a new program/initiative which is being piloted only in some clinics in Khayelitsha. In other clinics and other areas, people still have to come to their clinic to take their treatment every single day for two years.

1. Did you know this before you started getting a supply of treatment?
   1. Yes
   2. No
2. Do you understand why **you** were given a supply of RR-TB treatment to take by yourself in your home?
   1. Yes
   2. No

Please elaborate on your response:

____________________________________________________________________________________________________________________________________________________________

1. Did you find it helpful for the RR-TB counselor (Buci) to talk to you in a counseling session before you were given a supply of treatment to take at home?
   1. Yes
   2. No

Please elaborate on your response:

______________________________________________________________________________

1. What were your concerns when the clinic first offered to give you a supply of your treatment to take at home?

______________________________________________________________________________

1. Did you find it helpful for the RR-TB counselor (Buci) to explain your treatment?
   1. Yes
   2. No

Please elaborate on your response:

______________________________________________________________________________

1. Did you find it helpful for the RR-TB counselor (Buci) to explain your pillbox?
   1. Yes
   2. No

Please elaborate on your response:

________________________________________________________________________

1. Did you find it helpful for the RR-TB counselor (Buci) to explain how to remember to take your treatment after you were given a supply to take at home?
   1. Yes
   2. No

Please elaborate on your response: ______________________________________________________________________________

1. Do you like getting a supply of treatment to take at home?
   1. Yes
   2. No

____________________________________________________________________________________________________________________________________________________________

10) What did you like about getting a supply of treatment to take at home?

______________________________________________________________________________

11) What did you dislike about getting a supply of treatment to take at home?

______________________________________________________________________________

12) Do you think **all** RR-TB patients should be given a supply of medications to take at home?

- 1. Yes
  2. No
  3. Not sure

Please elaborate on your response: ______________________________________________________________________________

**Section 2: Adherence**

**The following questions should be answered in respect to taking your treatment**

1. Are you comfortable with taking your treatment at home with support from others?
   1. Yes
   2. No
   3. Not sure

Please elaborate on your response: ­­­­­­­­­­­­­­­­­­­_____________________________________________________________

1. Would you find it easier to take your treatment in the clinic every day, rather than at home?
   1. Yes
   2. No
   3. Not sure

Please elaborate on your response: ­­­­­­­­­­­­­­­­­­­_____________________________________________________________

**Prompt:** Everybody forgets to take their treatment sometimes, nobody is perfect.

1. How often do you forget to take your treatment?
   1. Never– I take it every single day of every week
   2. Sometimes – some days it is difficult to remember to take my treatment
   3. Always – it is very difficult to remember to take my treatment every day

If you answered sometimes or always, please provide reasons why you forget to take your treatment? (If difficult to answer, give suggestions, e.g. forgot where it is kept in the house, no alarm clock, have to go to work early and nowhere to keep meds, children distract me, etc)

_______________________________________________

_______________________________________________

_______________________________________________

1. Are there times where you remember your treatment but cannot take it for some reason (e.g. unexpected events where you find yourself without pills near you)?
   1. Never – I take it every single time I remember to take it
   2. Sometimes – some days it is difficult to take my treatment even though I remember to take it
   3. Always – it is very difficult to take my treatment every day even though I remember to take it

If you answered sometimes or always, please provide examples of situations where you didn’t take your treatment, even though you remembered to take it.

_______________________________________________

_______________________________________________

_______________________________________________

1. What kinds of things make it especially difficult to take your treatment? (If difficult to answer, give suggestions, e.g. drinking alcohol, going away on weekends, family emergencies, no money for food, makes me feel bad, etc)

______________________________________________

_______________________________________________

_______________________________________________

1. What helps you to remember to take your treatment every day? (If difficult to answer, give suggestions, e.g. stickers around house, phone, children, my three reasons for living, etc)

______________________________________________

_______________________________________________

_______________________________________________

**Section 3: The CCW’s**

**The following questions should be answered in respect to the CCW:**

1. The CCW checks in with me … (choose one option below)
   1. Never – I don’t manage to see the CCW every week/month
   2. Sometimes – I see the CCW some weeks/months but not others
   3. Always – I see the CCW every single week/month
2. The CCW checks my patient card… (choose one option below)
   1. Never– the CCW doesn’t see my card
   2. Sometimes – the CCW sees my card some times but not others
   3. Always – the CCW always sees my card
3. The CCW checks my pillbox … (choose one option below)
   1. Never – the CCW doesn’t check my pillbox
   2. Sometimes – the CCW checks my pillbox sometimes but not others
   3. Always– the CCW always checks my pillbox
4. Do you feel comfortable talking with the CCW who has been assigned to you?
   1. Yes
   2. No
   3. Not sure

Please elaborate on your response: _________________________________________________

1. Do you find it useful for the CCW to visit you at home once a week/month?
   1. Yes
   2. No
   3. Not sure

Please elaborate on your response: _________________________________________________

1. Do you think the CCW supports you?
   1. Yes
   2. No
   3. Not sure

Please elaborate on your response: _________________________________________________

1. Do you think the CCW helps you to stick to your treatment?
   1. Yes
   2. No
   3. Not sure

Please elaborate on your response: _________________________________________________

1. Do you feel like the CCW listens to you?
   1. Yes
   2. No
   3. Not sure

Please elaborate on your response: _________________________________________________

1. Do you think the CCW is understanding of your problems?
   1. Yes
   2. No
   3. Not sure

Please elaborate on your response: _________________________________________________

1. Do you think the CCW provides you with support similar to that you received at the clinic?
   1. Yes
   2. No
   3. Not sure

Please elaborate on your response: _________________________________________________

1. How do you think the CCW support could be improved?

____________________________________________________________________________________________________________________________________________________________

**Section 4: Self-Administered Treatment (SAT) Pilot Programme Overview**

**The following questions should be answered in respect to the overall SAT pilot programme**

1. Are you satisfied with the self-administered treatment pilot programme (getting a supply of treatment to take at home with support from a CCW) in your clinic?
   1. Yes
   2. No
   3. Not sure

Please elaborate on your response: _________________________________________________

1. Do you think the self-administered treatment pilot programme in your clinic is helpful for patients to complete their RR-TB treatment?
   1. Yes
   2. No
   3. Not sure

Please elaborate on your response: _________________________________________________

1. Has the self-administered treatment pilot programme in your clinic made it easier for people to take RR-TB treatment?
   1. Yes
   2. No
   3. Not sure

Please elaborate on your response: _________________________________________________

1. Do you receive additional support to take your RR-TB treatment?
   1. Yes
   2. No

Please elaborate on your response: _________________________________________________

1. What do you like about the self-administered treatment pilot programme in your clinic?

____________________________________________________________________________________________________________________________________________________________

1. What suggestions do you have to improve the self-administered treatment pilot programme in your clinic?

____________________________________________________________________________________________________________________________________________________________

1. Additional Comments:

____________________________________________________________________________________________________________________________________________________________

____________________________________________________________________________________________________________________________________________________________

**Section 5: Interviewer’s Notes**

**This section is to be filled out only by the independent interviewer (person administering the questionnaire).**

If you have any other comments about issues raised by the patient, that were not addressed above, please describe them here.

­­­­­­­­­­­­­_____________________________________________________________________________________

_____________________________________________________________________________________

_____________________________________________________________________________________

_____________________________________________________________________________________

_____________________________________________________________________________________

**Patient Satisfaction Questionnaire (isiXhosa)**

**Iphepha-mibuzo lolwaneliseko lwesigulane**

**Isifo sephepha esingayivayo i-Rifampicin (RR-TB) INkqubo yoLingo yoNyango ozinika ngoKwakho**

Ikliniki: ___________________

Inombolo yesazisi yesigulane: __________________________________

Ubudala: _________ Isini: Indoda ____ Umfazi ____Umhla: _________________

**Icandelo 1: Ukwaziswa kwiNkqubo yokuzinika uNyango (SAT)**

**L e mibuzo ilandelayo yimibuzo jikelele ngeNkqubo eLingwayo yokuzinika uNyango (SAT)**

1) Ingaba ufumana amayeza eveki okanye awenyanga onyango lwe-RR-TB kwiklliniki yakho ukuze uwatye ekhaya?

a. Ngeveki

b. Ngenyanga

c. Okunye: _____________________________________________________________

**Inkcazelo:** Ukunika abantu amayeza onyango lwe-RR-TB ukuze bawatyele emakhaya yinkqubo/ivelatanci elitsha nelilingwayo kwiikliniki ezithile zalapha eKhayelitsha. Kwezinye iikliniki nakwezinye iindawo, abantu kusafuneka beze kwiikliniki zabo yonke imihla ukuze batye amayeza abo iminyaka emibini.

2) Ubukwazi oku phambi kokuba uqalise ukufumana amayeza onyango?

a. Ewe

b. Hayi

3) Uyaqonda ukuba kutheni wanikwa amayeza onyango lwe RR-TB ukuba ugoduke nawo uye kuzityela ngokwakho ekhaya?

a. Ewe

b. Hayi

Nceda ucacise impendulo yakho:

______________________________________________________________________________

______________________________________________________________________________

4) Wakufumana kuluncedo na ukuba umcebisi wakho we-RR-TB (uBuci) athethe nawe kwiseshoni yokucebisa phambi kokuba unikwe amayeza onyango ukuze uwatye ekhaya?

a. Ewe

b. Hayi

Nceda ucacise impendulo yakho:

______________________________________________________________________________

______________________________________________________________________________

5) Yayiziintoni iinkxalabo zakho ukuqala kwekliniki ukukuthembisa ngokukunika amayeza onyango lwakho ukuze uwatye ekhaya?

______________________________________________________________________________

6) Wakufumana kuluncedo ukuba umcebisi wakho we-RR-TB (uBuci) acacise unyango lwakho?

a. Ewe

b. Hayi

Nceda ucacise impendulo yakho:

______________________________________________________________________________

______________________________________________________________________________

7) Ukufumene kuluncedo ukuba umcebisi we-RR-TB (uBuci) acacise ngebhokisi yakho yeepilisi?

a. Ewe

b. Hayi

Nceda ucacise impendulo yakho:

______________________________________________________________________________

______________________________________________________________________________

8) Ukufumene kuluncedo ukuba umcebisi we-RR-TB (uBuci) acacise ukuba ungakhumbula njani ukuba utye amayeza akho emva kokuba unikwe amayeza ukuze uwatye ekhaya?

a. Ewe

b. Hayi

Nceda ucacise impendulo yakho:

______________________________________________________________________________

______________________________________________________________________________

9) Uyathanda ukunikwa isixa samayeza uwatyele ekhaya?

a. Ewe

b. Hayi

10) Yintoni owayithandayo ngokunikwa amayeza ukuze uwatyele ekhaya?

______________________________________________________________________________

11) Yintoni ongazange uyithande ngokunikwa amayeza ukuze uwatyele ekhaya?

______________________________________________________________________________

12) Ucinga ukuba zonke izigulane ze-RR-TB kufuneka zinikwe amayeza ukuze ziwatyele emakhaya?

a. Ewe

b. Hayi

c.Andiqinisekanga

Nceda ucacise impendulo yakho:

______________________________________________________________________________

______________________________________________________________________________

**Icandelo 2: Ukuthobela**

**Le mibuzo ilandelayo kufuneka iphendulwe ngokuphathelele nokutya amayeza akho**

1) Uyakonwabela ukutya amayeza akho ekhaya ngenkxaso yabanye?

a. Ewe

b. Hayi

c. Akaqinisekanga

Nceda ucacise impendulo yakho:

________________________________________________________________________

2) Ungakufumana kulula ukutya amayeza akho ekliniki yonke imihla, kunokuwatyela ekhaya?

a. Ewe

b. Hayi

c. Akaqinisekanga

Nceda ucacise impendulo yakho:

________________________________________________________________________

**Inkcaza:** Wonke umntu uyalibala ukutya amayeza akhe ngamanye amaxesha, akukho ugqibeleleyo.

3) Ingaba ulibala kangakanani ukutya amayeza akho?

a. Andizange– Ndiwatya yonke imihla zonke iiveki

b. Ngamanye amaxesha – ngezinye iintsuku kunzima ukukhumbula ukutya amayeza am

c. Rhoqo – kunzima kakhulu ukukhumbula ukutya amayeza am yonke imihla

Ukuba impendulo yakho ithe ngamanye amaxesha okanye rhoqo, nceda unike izizathu zokuba ulibale ukutya amayeza akho? (ukuba kunzima ukuphendula, nika umzekelo: ulibele apho abekwa khona endlwini, akukho wotshi ikhalayo, kufuneka ndiye emsebenzini kwakusasa yaye andinandawo yakugcina mayeza, abantwana bayandiphazamisa, njl.njl.)

___________________________________________________________________________

___________________________________________________________________________

___________________________________________________________________________

4) Ingaba akhona amaxesha apho ukhumbula ngawo amayeza akho kodwa ungenako ukuwatya ngennxa yesizathu esithile (umz.

iziganeko ezingalindelekanga apho ufumana kungekho zipilisi kufutshane nawe)?

a. Azange – Ndiyitya qho ndikhumbula ukuyitya

b. Ngamanye amaxesha – ngezinye iintsuku kunzima ukutya amayeza am nangona ndikukhumbule ukuwatya

c. Rhoqo – kunzima kakhulu ukutya amayeza am yonke imihla nangona ndikukhumbula ukuwatya

Ukuba impendulo yakho ithe ngamanye amaxesha okanye rhoqo, nceda unike imizekelo eemeko apho ungazange uwatye amayeza akho, nangona ukukhumbule ukuwatya.

___________________________________________________________________________

___________________________________________________________________________

___________________________________________________________________________

5) Loluphi olona hlobo lwezinto ezenza kube nzima ngakumbi ukutya amayeza akho? (Ukuba kunzima ukuphendula nika umzekelo: ukusela utywala, ukuhamba ngeempelaveki, izinto ezingxamisekileyo zosapho, ukungabikho kwemali yokutya, kundenza ndizive kakubi, njl.njl.)

___________________________________________________________________________

___________________________________________________________________________

___________________________________________________________________________

6) Yintoni ekunceda ukuba ukhumbule ukutya amayeza akho yonke imihla? (ukuba kunzima ukuphendula, nika iingcebiso, umz. izinto ezincanyathiselwe endlwini, ifowni, abantwana, izizathu zam ezithathu zokuphila, njl.njl.)

___________________________________________________________________________

___________________________________________________________________________

___________________________________________________________________________

**Icandelo 3: Ii-CCW**

**Le mibuzo ilandelayo kufuneka iphendulwe ngokuphathelele kwi-CCW:**

1) I-CCW iyandivelela … (khetha ibe nye ngezantsi)

a. Ayizange – Andikwazi ukudibana ne-CCW qho ngeveki/ngenyanga

b. Ngamanye amaxesha – ndiyadibana ne-CCW ngezinye iiveki/iinyanga

c. Rhoqo – Ndidibana ne-CCW qho ngeveki/ngenyanga

2) I-CCW iyalijonga ikhadi lam lekliniki… (khetha ibe nye ngezantsi)

a. Ayizange– i-CCW ayiliboni ikhadi lam

b. Ngamanye amaxesha – i-CCW iyalijonga ikhadi lam kodwa hayi ngamanye

c. Rhoqo – i-CCW isoloko ilijonga ikhadi lam

3) I-CCW iyayijonga ibhokisi yam yeepilisi … (khetha ibe nye ngezantsi)

a. Ayizange – i-CCW ayiyijongi ibhokisi yam yeepilisi

b. Ngamanye amaxesha – i-CCW ijonga ibhokisi yam yeepilisi ngamanye amaxesha

c. Rhoqo– I-CCW isoloko ijonga ibhokisi yam yeepilisi

4) Uziva ukhululekile ukuthetha ne-CCW owabelwe yona?

a. Ewe

b. Hayi

c. Andiqinisekanga

Nceda ucacise impendulo yakho: _________________________________________________

5) Ukufumana kuluncedo ukuba i-CCW ikundwendwele ekhaya kanye ngeveki/nyanga?

a. Ewe

b. Hayi

c. Andiqinisekanga

Nceda ucacise impendulo yakho: _________________________________________________

6) Ucinga ukuba i-CCW iyakuxhasa?

a. Ewe

b. Hayi

c. Andiqinisekanga

Nceda ucacise impendulo yakho: _________________________________________________

7) Ucinga ukuba i-CCW iyakunceda ungohlukani namayeza akho?

a. Ewe

b. Hayi

c. Andiqinisekanga

Nceda ucacise impendulo yakho: _________________________________________________

8) Uva ngathi i-CCW iyakumamela?

a. Ewe

b. Hayi

c. Andiqinisekanga

Nceda ucacise impendulo yakho: _________________________________________________

9) Ucinga ukuba i-CCW iyaziqonda iingxaki zakho?

a. Ewe

b. Hayi

c. Andiqinisekanga

Nceda ucacise impendulo yakho: _________________________________________________

10) Ucinga ukuba i-CCW ikunika inkxaso efanayo naleyo ubuyifumana ekliniki?

a. Ewe

b. Hayi

c. Andiqinisekanga

Nceda ucacise impendulo yakho: _________________________________________________

11) Ucinga ukuba ingaphuculwa njani inkxaso ye-CCW?

______________________________________________________________________________

______________________________________________________________________________

**Icandelo 4: Isishwankathelo seNkqubo eLingwayo yoKuzinika uNyango (SAT)**

**Le mibuzo ilandelayo kufuneka iphendulwe ngokuphathelele kwinkqubo elingwayo ye-SAT xa iyonke**

1) Ingaba wanelisekile yinkqubo elingwayo yokuzinika unyango (ukufumana amayeza ukuze uwatye ekhaya ngenkxaso ye-CCW) kwikliniki yakho?

a. Ewe

b. Hayi

c. Andiqinisekanga

Nceda ucacise impendulo yakho: _________________________________________________

2) Ingaba ucinga ukuba inkqubo elingwayo yokuzinika unyango kwikliniki yakho iluncedo ukuba izigulane zigqibe ukutya amayeza azo e-RR-TB?

a. Ewe

b. Hayi

c. Andiqinisekanga

Nceda ucacise impendulo yakho: _________________________________________________

3) Ingaba inkqubo elingwayo yokuzinika unyango kwikliniki yakho yenze kwaba lula ebantwini ukutya amayeza e-RR-TB?

a. Ewe

b. Hayi

c. Andiqinisekanga

Nceda ucacise impendulo yakho: _________________________________________________

4) Ingaba ufumana inkxaso eyongezelelweyo ukuze utye amayeza akho e-RR-TB?

a. Ewe

b. Hayi

Nceda ucacise impendulo yakho: _________________________________________________

5) Yintoni oyithandayo ngenkqubo elingwayo yonyango ozinika ngokwakho kwikliniki yakho?

______________________________________________________________________________

______________________________________________________________________________

6) Ziziphi iimbono onazo zokuphucula inkqubo elingwayo yonyango ozinika ngokwakho kwikliniki yakho?

______________________________________________________________________________

______________________________________________________________________________

7) Iintetho ezongezelelweyo:

______________________________________________________________________________

______________________________________________________________________________

I**candelo 5: Amanqaku alowo ubuza imibuzo:**

**Eli candelo kufuneka lizaliswe kuphela ngumbuzi mibuzo ozimeleyo (Umntu osebenzisa iphepha-mibuzo).**

Ukuba unazo ezinye iintetho malunga nemiba ephakanyiswe sisigulane engakhange iqwalaselwe ngentla, nceda uyichaze apha.

__________________________________________________________________________________

__________________________________________________________________________________

__________________________________________________________________________________

__________________________________________________________________________________

__________________________________________________________________________________

**Health Care Worker (HCW) Satisfaction Questionnaire (English)**

**Rifampicin-Resistant Tuberculosis (RR-TB) Self-Administered Treatment (SAT) Pilot Programme**

HCW Identification Number: ____________________________________

Age: ____________________ Sex: Male ____ Female ____ Clinic: ___________________

Length of time working at clinic: _______________ Date: _________________

The self-administered treatment (SAT) pilot programme is being conducted in eight different clinics within Khayelitsha with the aim of determining whether it is feasible for patients to self-administer treatment outside of the clinic without directly observed therapy (DOT). The pilot programme was implemented in 2011 by Medecins Sans Frontieres (MSF), a medical humanitarian organization which has been involved in the provision of decentralized rifampicin-resistant tuberculosis (RR-TB) care in Khayelitsha since 2007.

The purpose of this questionnaire is to ascertain information on your perspectives regarding the SAT pilot programme to date to help us evaluate the programme and improve our services in future.

**Section 1: SAT Overview**

1. In what ways does the SAT pilot programme differ from the standard of care? (examples: self-administration of treatment, treatment in the community, no DOT, monthly clinic visits)

**____________________________________________________________________________________________________________________________________________________________**

1. What do you think the main objectives of the SAT pilot programme are?

**____________________________________________________________________________________________________________________________________________________________**

1. **­­­­­­­­­­­**When do patients become eligible for the SAT pilot programme?

**____________________________________________________________________________________________________________________________________________________________**

1. Do you think the eligibility criteria should be changed?
   1. Yes
   2. No

Please elaborate on your response:  **____________________________________________________________________________________________________________________________________________________________**

1. What role do you think the RR-TB counselors play in the SAT pilot programme?

**____________________________________________________________________________________________________________________________________________________________**

1. What role do you think the Community Care Workers (CCWs) play in the SAT pilot programme?

**____________________________________________________________________________________________________________________________________________________________**

1. What role do you as a Health Care Worker (HCW) play in the SAT pilot programme?

**____________________________________________________________________________________________________________________________________________________________**

1. How often do you see RR-TB patients who have been placed out in the SAT pilot programme?

**____________________________________________________________________________________________________________________________________________________________**

1. What do you do if you find out that one of your patients placed out on the SAT pilot programme is having trouble taking treatment at home every day?

**____________________________________________________________________________________________________________________________________________________________**

**Section 2. Perspectives**

1. Do you think the SAT pilot programme has assisted RR-TB patients in adhering to their treatment?
   1. Yes
   2. No

Please elaborate on your response (examples):

**____________________________________________________________________________________________________________________________________________________________**

1. Do you think the SAT pilot programme relieved pressure on the clinic?
   1. Yes
   2. No

Please elaborate on your response (examples: fewer patients coming to the clinics routinely, more time to spend with complicated cases).

**____________________________________________________________________________________________________________________________________________________________**

1. Do you think the SAT pilot programme is useful for RR-TB patients?
   1. Yes
   2. No

Please elaborate on your response:

**____________________________________________________________________________________________________________________________________________________________**

1. Do you think the RR-TB counselors play an important role when patients are assessed for placement onto the SAT pilot programme?
   1. Yes
   2. No

Please elaborate on your response:

**____________________________________________________________________________________________________________________________________________________________**

1. Why do you think counselors are important in the SAT pilot programme?

**____________________________________________________________________________________________________________________________________________________________**

1. Do you think the CCWs play an important role in the SAT pilot programme for RR-TB patients?
   1. Yes
   2. No

Please elaborate on your response:

**____________________________________________________________________________________________________________________________________________________________**

1. In what ways do the CCWs assist patients in taking their treatment once enrolled in the SAT pilot programme?

**____________________________________________________________________________________________________________________________________________________________**

1. Do you think adherence has improved among patients enrolled in the SAT pilot programme?
   1. Yes
   2. No

Please elaborate on your response:

**____________________________________________________________________________________________________________________________________________________________**

1. Have any of your RR-TB patients struggled with the SAT pilot programme?
   1. Yes
   2. No

Please elaborate on your response:

**____________________________________________________________________________________________________________________________________________________________**

1. What are the main challenges? Please provide three examples.
   1. __________
   2. __________
   3. __________
2. Have you faced challenges providing care to RR-TB patients enrolled in the SAT pilot programme?
   1. Yes
   2. No

Please elaborate on your response:

**___________________________________________________________________________________________________________________________________________________________**

1. What are the three main benefits of the RR-TB SAT pilot programme for you as a HCW?
   1. __________
   2. __________
   3. __________
2. What are the three main benefits of the SAT pilot programme for the RR-TB patients?
   1. __________
   2. __________
   3. __________
3. What are the three main benefits of the SAT pilot programme for the CCWs?
   1. __________
   2. __________
   3. __________
4. What do you currently like best about the SAT pilot programme for RR-TB patients?

**____________________________________________________________________________________________________________________________________________________________**

1. What are your concerns about the SAT pilot programme for RR-TB patients?

**____________________________________________________________________________________________________________________________________________________________**

1. Is there anything you would like to change about the SAT pilot programme?

**____________________________________________________________________________________________________________________________________________________________**

**Health Care Worker (HCW) Satisfaction Questionnaire (isiXhosa)**

**Iphepha-mibuzo loLwanesliseko loMsebenzi woNonophelo lweMpilo (HWC)**

**INkqubo eLingwayo yokuZinika uNyango (SAT) lweSifo sePhepha esingaYivayo i-Rifampicin (RR-TB)**

Inombolo yeSazisi yeHWC: ______________________

Ubudala: ____________________ Isini: Indoda ____ Umfazi ____

Ikliniki: ___________________

Ubude bexesha esebenza ekliniki: _______________ Umhla: _________________

Le nkqubo ilingwayo yokuzinika unyango (SAT) iqhutywa kwiikliniki ezisibhozo ezahlukeneyo kummandla waseKhayelitsha ngenjongo yokuqwalasela ukuba kungenzeka na ukuba izigulane zizinike ngokwazo unyango ngaphandle kweekliniki ngaphandle konyango olunonotshelwa ngqo (DOT). Le nkqubo ilingwayo yamiliselwa ngowama 2011 ngabeMedecins Sans Frontieres (MSF), umbutho wobuntu wamayeza obusoloko ubandakanyeka ekuboneleleni ngononophelo olungazinzanga lwesifo sephepha esingayivayo i-rifampicin (RR-TB) eKhayelitsha ukususela ngowama 2007.

Injongo yeli phepha-mibuzo kukuqinisekisa ulwazi ngezimvo zenu malunga nenkqubo elingwayo yeSAT ukuza kuthi ga ngoku ukusinceda sixabise le nkqubo yaye siphucule iinkonzo zethu kwixa elizayo.

**Icandelo 1: Isishwankathelo seNkqubo eLingwayo yokuzinika uNyango (SAT)**

1) Ziziphi iindlela inkqubo elingwayo iSAT eyahluke ngazo kunonophelo lwesiqhelo? (imizekelo: ukuzinika unyango, unyango ekuhlaleni, akukho DOT, ukundwendwela ekliniki qho ngenyanga)

______________________________________________________________________________

______________________________________________________________________________

2) Ucinga ukuba yintoni ezona njongo zenkqubo elingwayo iSAT?

______________________________________________________________________________

______________________________________________________________________________

3) Ziyilungela nini izigulane inkqubo elingwayo iSAT?

______________________________________________________________________________

______________________________________________________________________________

4) Ucinga ukuba iimfuno zokulungela kufuneka zitshintshwe?

a. Ewe

b. Hayi

Nceda ucacise impendulo yakho:

______________________________________________________________________________

______________________________________________________________________________

5) Yeyiphi indima ocinga ukuba abacebisi be-RR-TB bayayidlala kwinkqubo elingwayo iSAT?

______________________________________________________________________________

______________________________________________________________________________

6) Yeyiphi indima ocinga ukuba bayayidlala aBasebenzi boNonophelo baseKuhlaleni (CCW) kwinkqubo elingwayo iSAT?

______________________________________________________________________________

______________________________________________________________________________

7) Yeyiphi indima wena njengoMsebenzi woNonophelo lweMpilo (HCW) oyidlalayo kwinkqubo elingwayo iSAT?

______________________________________________________________________________

______________________________________________________________________________

8) Uzibona kangakanani izigulane ze-RR-TB ezikhutshelwe kwinkqubo elingwayo iSAT?

______________________________________________________________________________

______________________________________________________________________________

9) Wenza ntoni xa ufumanisa ukuba esinye sezigulane zakho ezikhutshelwe kwinkqubo elingwayo iSAT sinengxaki yokutya amayeza ekhaya yonke imihla?

______________________________________________________________________________

______________________________________________________________________________

**Icandelo 2. Izimvo**

1) Ucinga ukuba inkqubo elingwayo iSAT izincedile izigulane ze-RR-TB ukuba zihlale zisitya amayeza azo?

a. Ewe

b. Hayi

Nceda ucacise impendulo yakho (imizekelo):

______________________________________________________________________________

______________________________________________________________________________

2) Ucinga ukuba inkqubo elingwayo iSAT iphungule uxinzelelo ekliniki?

a. Ewe

b. Hayi

Nceda ucacise impendulo yakho (imizekelo: zimbalwa izigulane eziza ezikliniki ngokufanelekileyo,

lininzi ixesha elichithwa kwiimeko ezimandundu).

______________________________________________________________________________

______________________________________________________________________________

3) Ucinga ukuba inkqubo elingwayo iSAT iluncedo kwizigulane ze-RR-TB?

a. Ewe

b. Hayi

Nceda ucacise impendulo yakho:

______________________________________________________________________________

______________________________________________________________________________

4) Ucinga ukuba abacebisi be-RR-TB badlala indima ebalulekileyo xa izigulane zihlolelwa ukulungela ukukhutshelwa kwinkqubo elingwayo iSAT?

a. Ewe

b. Hayi

Nceda ucacise impendulo yakho:

______________________________________________________________________________

______________________________________________________________________________

5) Kutheni ucinga ukuba abaceisi babalulekile kwinkqubo elingwayo iSAT?

______________________________________________________________________________

______________________________________________________________________________

6) Ucinga ukuba zibalulekile ii-CCW kwinkqubo elingwayo iSAT yezigulane ze-RR-TB?

a. Ewe

b. Hayi

Nceda ucacise impendulo yakho:

______________________________________________________________________________

______________________________________________________________________________

7) Ziziphi iindlela ocinga ukuba ii-CCW zinceda ngazo izigulane ekutyeni amayeza azo zakube zifakwe kwinkqubo elingwayo iSAT?

______________________________________________________________________________

______________________________________________________________________________

8) Ucinga ukuba ukuthobela kuphucukile kwizigulane ezifakwe kwinkqubo elingwayo iSAT?

a. Ewe

b. Hayi

Nceda ucacise impendulo yakho:

______________________________________________________________________________

______________________________________________________________________________

9) Ingaba sikhona kwizigulane zakho ze-RR-TB esisokoliswe yinkqubo elingwayo iSAT?

a. Ewe

b. Hayi

Nceda ucacise impendulo yakho:

______________________________________________________________________________

______________________________________________________________________________

10) Yeyiphi eyona mingeni? Nceda unike imizekelo emithathu.

a. ________________________________________________

b. ________________________________________________

c. ________________________________________________

11) Ukhe wajongana nemingeni na ekuboneleleni ngononophelo kwizigulane ze-RR-TB ezifakwe kwinkqubo elingwayo iSAT?

a. Ewe

b. Hayi

Nceda ucacise impendulo yakho:

______________________________________________________________________________

_____________________________________________________________________________

12) Ziziphi ezona zinto zintathu zixhamlekayo kwinkqubo elingwayo i-RR-TB SAT kuwe njenge-HCW?

a. _________________________________________________

b. _________________________________________________

c. _________________________________________________

13) Ziziphi ezona zinto zintathu zixhamlekayo kwinkqubo elingwayo iSAT kwizigulane ze-RR-TB?

a. _________________________________________________

b. _________________________________________________

c. _________________________________________________

14) Ziziphi ezona zinto zintathu zixhamlekayo kwinkqubo elingwayo iSAT kwii-CCW?

a. _________________________________________________

b. _________________________________________________

c. _________________________________________________

15) Yintoni kungokunje oyithanda kakhulu ngenkqubo elingwayo iSAT kwizigulane ze-RR-TB?

______________________________________________________________________________

______________________________________________________________________________

16) Ziintoni iinkxalabo zakho malunga nenkqubo elingwayo iSAT kwizigulane ze-RR-TB?

______________________________________________________________________________

______________________________________________________________________________

17) Ikhona into onqwenela ukuyitshintsha malunga nenkqubo elingwayo iSAT?

______________________________________________________________________________

______________________________________________________________________________

**Community Care Worker (CCW) Satisfaction Questionnaire (English)**

**Rifampicin-Resistant Tuberculosis (RR-TB) Self-Administered Treatment (SAT) Pilot Programme**

CCW Identification Number:

Age: __________________ Sex: Male _____ Female ____

Clinic: ___________________

Length of time working at clinic: _______________ Date: _________________

The self-administered treatment (SAT) pilot programme is being conducted in eight different clinics within Khayelitsha with the aim of determining whether it is feasible for patients to self-administer treatment outside of the clinic without directly observed therapy (DOT). The pilot programme was implemented in 2011 by Medecins Sans Frontieres (MSF), a medical humanitarian organization which has been involved in the provision of decentralized rifampicin-resistant tuberculosis (RR-TB) care in Khayelitsha since 2007.

The purpose of this questionnaire is to ascertain information on your perspectives regarding the SAT pilot programme to date to help us evaluate the programme and improve our services in future.

**Section 1: CT Overview**

1. In what ways does the SAT pilot programme differ from the standard of care? (examples: self-administration of treatment, treatment in the community, no DOT, monthly clinic visits)

**____________________________________________________________________________________________________________________________________________________________**

1. What do you think the main objectives of the SAT pilot programme are?

**____________________________________________________________________________________________________________________________________________________________**

1. **­­­­­­­­­­­**When do patients become eligible for the SAT pilot programme?

**____________________________________________________________________________________________________________________________________________________________**

1. Do you think the eligibility criteria should be changed?
   1. Yes
   2. No

Why?

**____________________________________________________________________________________________________________________________________________________________**

1. What role do you think the RR-TB counselors play in the SAT pilot programme?

**____________________________________________________________________________________________________________________________________________________________**

1. What role do you think the Health Care Workers (HCWs) (i.e. DR-TB nurses) play in the SAT pilot programme?

**____________________________________________________________________________________________________________________________________________________________**

1. What role do you, as the Community Care Worker (CCW), play in the SAT pilot programme?

**____________________________________________________________________________________________________________________________________________________________**

1. How often do you visit your RR-TB patients at home?

**____________________________________________________________________________________________________________________________________________________________**

1. Do you struggle to visit your RR-TB patients at home?
   1. Yes
   2. No

If yes, provide examples of why you struggle to see your patients.  **_____________________________________________________________________________________ _______________________________________________________________________**

1. Do you check the RR-TB patients’ pill box on every visit?
   1. Yes
   2. No
2. What do you do if you notice your patient is having trouble taking his/her medications?

**____________________________________________________________________________________________________________________________________________________________**

**Section 2. Perspectives**

1. Do you think the SAT pilot programme has assisted RR-TB patients in adhering to their treatment?
   1. Yes
   2. No

Please elaborate on your response:

**____________________________________________________________________________________________________________________________________________________________**

1. Do you think the SAT pilot programme has made treating RR-TB patients easier for HCWs (RR-TB nurses)?
   1. Yes
   2. No

Please elaborate on your response:

**____________________________________________________________________________________________________________________________________________________________**

1. Do you think the SAT pilot programme is useful for RR-TB patients??
   1. Yes
   2. No

Please elaborate on your response:

**____________________________________________________________________________________________________________________________________________________________**

1. Do you think the RR-TB counselors play an important role when patients are assessed for placement into the SAT pilot programme?
   1. Yes
   2. No

Please elaborate on your response:

**____________________________________________________________________________________________________________________________________________________________**

1. Do you think adherence has improved among patients enrolled in the SAT pilot programme?
   1. Yes
   2. No

Please elaborate on your response:

**____________________________________________________________________________________________________________________________________________________________**

1. Do your RR-TB patients have problems taking their treatment every day at home?
   1. Yes
   2. No

Please elaborate on your response:

**____________________________________________________________________________________________________________________________________________________________**

1. What are the main challenges patient’s encounter in taking their treatment at home? Please provide three examples.
   1. __________
   2. __________
   3. __________
2. Do you face challenges providing care to your RR-TB patients enrolled in the SAT pilot programme?
   1. Yes
   2. No

Please elaborate on your response:

**___________________________________________________________________________________________________________________________________________________________**

1. What are the three main benefits of the SAT pilot programme for you as a CCW?
   1. __________
   2. __________
   3. __________
2. What are the three main benefits of the SAT pilot programme for the RR-TB patients?
   1. __________
   2. __________
   3. __________
3. What are the three main benefits of the SAT pilot programme for Health Care providers for example nurses and/or medical officers?
   1. __________
   2. __________
   3. __________
4. What do you currently like best about the SAT pilot programme?

**____________________________________________________________________________________________________________________________________________________________**

1. Do you believe the support you give to RR-TB patients is different than that the support they would receive if they had continued to receive DOT in their clinic every day?
   1. Yes
   2. No

Please elaborate on your response and provide specific examples:

**____________________________________________________________________________________________________________________________________________________________**

1. What are your concerns about the SAT pilot programme?

**____________________________________________________________________________________________________________________________________________________________**

1. Is there anything you would like to change about the SAT pilot programme?

**____________________________________________________________________________________________________________________________________________________________**

**Community Care Worker (CCW) Satisfaction Questionnaire (isiXhosa)**

**Iphepha-mibuzo loMsebenzi woNonophelo waseKuhlaleni (CCW)**

**INkqubo eLingwayo yokuZinika uNyango (SAT) lweSifo sePhepha esingayivayo i-lRifampicin (RR-TB)**

Inombolo yeSazisi ye-CCW: _____________________________

Ubudala: __________________ Isini: Indoda _____ Umfazi ____

Ikliniki: ___________________

Ubude bexesha esebenza ekliniki: _______________ Umhla: _________________

Inkqubo elingwayo yokuzinika unyango (SAT) iqhutywa kwiikliniki ezisibhozo ezahlukeneyo eKhayelitsha ngenjongo yokuqwalasela ukuba kungenzeka na izigulane zizinike ngokwazo onyango ngaphandle kwekliniki ngaphandle konyango olunonotshelwe ngqo (DOT). le nkqubo ilingwayo yamiliselwa ngowama 2011 ngabeMedecins Sans Frontieres (MSF), umbutho wobuntu wamayeza obusoloko ubandakanyeka ekuboneleleni ngononophelo olungazinzanga lwesifo sephepha esingayivayo i-rifampicin (RR-TB) eKhayelitsha ukususela ngowama 2007.

Injongo yeli phepha-mibuzo kukuqinisekisa ulwazi ngezimvo zenu malunga nenkqubo elingwayo i-SAT ukuza kuthi ga ngoku ukusinceda sixabise le nkqubo siphucule iinkonzo zethu kwixa elizayo.

**Icandelo 1: Isishwankathelo seNkqubo eLingwayo yokuZinika uNyango (SAT)**

1.Zeziphi iindlela eyahluke ngazo inkqubo elingwayo iSAT kunonophelo lwesiqhelo? (imizekelo: ukuzinika unyango ngokwakho, unyango ekuhlaleni, akukho DOT, ukundwendwela ekliniki qho ngenyanga)

______________________________________________________________________________

______________________________________________________________________________

2. Ucinga ukuba ziyintoni ezona njongo ziphambili zenkqubo elingwayo i-SAT?

______________________________________________________________________________

______________________________________________________________________________

3. Ziyilungela nini izigulane inkqubo elingwayo iSAT?

______________________________________________________________________________

______________________________________________________________________________

4. Ucinga ukuba iimfuno zokulungela kufuneka zitshintshwe?

a. Ewe

b. Hayi

Ngoba?

______________________________________________________________________________

______________________________________________________________________________

5. Yiyiphi indima ocinga ukuba idlalwa ngabacebisi be-RR-TB kwinkqubo elingwayo iSAT?

______________________________________________________________________________

______________________________________________________________________________

6. Yiyiphi indima ocinga ukuba aBasebenzi boNonophelo lweMpilo (HCW) (okt. abongikazi be-RR-TB) bayayidlala kwinkqubo elingwayo iSAT?

__________________________________________________________________________________

__________________________________________________________________________________

7. Yiyiphi indima wena, njengoMsebenzi woNonophelo lweMpilo (CCW), oyidlalayo kwinkqubo elingwayo iSAT?

______________________________________________________________________________

______________________________________________________________________________

8. Uzityelela kangakanani izigulane zakho ze-RR-TB emakhaya?

______________________________________________________________________________

______________________________________________________________________________

9. Uyasokola ukutyelela izigulane zakho ze-RR-TB emakhaya?

a. Ewe

b. Hayi

Ukuba kunjalo, nika imizekelo yokuba kutheni usokola ukubona izigulane zakho.

______________________________________________________________________________

_______ _______________________________________________________________________

10. Uyayihlola ibhokisi yeepilisi yesigulane se-RR-TB qho utyelela?

a. Ewe

b. Hayi

11. Wenza ntoni xa uqaphela ukuba isigulane sakho sinengxaki yokutya amayeza aso?

______________________________________________________________________________

______________________________________________________________________________

Icandelo 2. Izimvo

1) Ucinga uuba inkqubo elingwayo iSAT izincedile izigulane ze-RR-TB ukuze zihlale zisitya amayeza azo?

a. Ewe

b. Hayi

Nceda ucacise impendulo yakho:

______________________________________________________________________________

______________________________________________________________________________

2) Ucinga ukuba inkqubo elingwayo i-SAT yenze ukuba kube lula ukunyanga izigulane ze-RR-TB kwii-HCW (abongikazi be-RR-TB)?

a. Ewe

b. Hayi

Nceda ucacise impendulo yakho:

______________________________________________________________________________

______________________________________________________________________________

3) Ucinga ukuba inkqubo elingwayo i-SAT iluncedo kwizigulane ze-RR-TB?

a. Ewe

b. Hayi

Nceda ucacise impendulo yakho:

______________________________________________________________________________

______________________________________________________________________________

4) Ucinga ukuba abacebisi be-RR-TB badlala indima ebalulekileyo xa izigulane zihlolelwa ukufakwa kwinkqubo elingwayo i-SAT?

a. Ewe

b. Hayi

Nceda ucacise impendulo yakho:

______________________________________________________________________________

______________________________________________________________________________

5) Ucinga ukuba kuphucukile ukuhlala zisitya amayeza izigulane ezifakwe kwinkqubo elingwayo i-SAT?

a. Ewe

b. Hayi

Nceda ucacise impendulo yakho:

______________________________________________________________________________

______________________________________________________________________________

6) Izigulane zakho ze-RR-TB ziba nengxaki yokutya amayeza azo yonke imihla emakhaya?

a. Ewe

b. Hayi

Nceda ucacise impendulo yakho:

______________________________________________________________________________

______________________________________________________________________________

7) Yeyiphi eyona mingeni imandla ezihlangabezana nayo izigulane ekutyeni amayeza azo emakhaya? Nceda unike imizekelo emithathu.

a. _________________________________________________________________

b. _________________________________________________________________

c. _________________________________________________________________

8) Uhlangabezana nemingeni ekuboneleleni ngononophelo kwizigulane zakho ze-RR-TB ezifakwe kwinkqubo elingwayo i-SAT?

a. Ewe

b. Hayi

Nceda ucacise impendulo yakho:

______________________________________________________________________________

______________________________________________________________________________

9) Ziziphi ezona zinto zimandla zintathu uzixhamlayo kwinkqubo elingwayo i-SAT wena njenge-CCW?

a. ____________________________________________________________

b. ____________________________________________________________

c. ____________________________________________________________

10) Ziziphi ezona zinto zintathu zimandla zixhanyulwa kwinkqubo elingwayo i-SAT zizigulane ze-RR-TB?

a. ______________________________________________________________

b. ______________________________________________________________

c. ______________________________________________________________

11) Ziziphi ezona zinto zintathu zimandla zixhanyulwa kwinkqubo elingwayo i-SAT ngababoneleli boNonophelo lweMpilo umzkelo abongikazi kunye/okanye amagosa empilo?

a. ______________________________________________________________

b. ______________________________________________________________

c. ______________________________________________________________

12) Yintoni kungokunje oyithanda kakhulu ngenkqubo elingwayo i-SAT?

______________________________________________________________________________

______________________________________________________________________________

13) Ucinga ukuba inkxaso oyinika izigulane ze-RR-TB yahlukile kwinkxaso ebeziya kuyifumana ukuba beziqhubile nokufumana i-DOT kwiikliniki zazo yonke imihla?

a. Ewe

b. Hayi

Nceda ucacise impendulo yakho unike nemizekelo ecacileyo:

______________________________________________________________________________

______________________________________________________________________________

14) Ziyintoni iinkxalabo zakho ngenkqubo elingwayo i-SAT?

______________________________________________________________________________

______________________________________________________________________________

15) Ikhona into onqwenela ukuyitshintsha ngenkqubo elingwayo i-SAT?

______________________________________________________________________________

______________________________________________________________________________
