# Supplementary material for: “Life continues”: Patient, health care and community care workers perspectives on self-administered treatment for rifampicin-resistant tuberculosis in Khayelitsha, South Africa
Source: PLoS One. 2018 Sep 14;13(9):e0203888. doi: 10.1371/journal.pone.0203888 (PMC6138394; doi:10.1371/journal.pone.0203888)
Supplement: S2 File — (DOCX) [file pone.0203888.s002.docx]

**Annex 2: In-depth interview guide for patients (English):
Self-administered treatment (SAT) pilot programme evaluation**

Thank you for agreeing to talk with me today, and for giving your informed consent. I would like to ask you about your experiences of taking part in the SAT pilot programme in Khayelitsha. All of your answers will be kept confidential, and you do not have to answer any questions that you don’t want to. There are no right or wrong answers to my questions. Please feel free to ask questions at any point during the discussion.

1. Can you explain to me what you know about the SAT pilot programme that you have been participating in?

**Probes:** understanding of programme, reasons for programme, patient inclusion criteria, own involvement

2. How did you feel when you were asked if you would like to take your RR-TB treatment home?

**Probes:** positive or negative feelings, concerns and questions, understanding reason for eligibility

3. What have been your experiences of taking your treatment at home?

**Probes:** ease/difficulties, change to routine, disclosure to others, length of supply, concerns

4. Can you explain to me if your daily routine has changed since you began taking your treatment at home? Can you explain to me what your days were like before the pilot, and during it?

**Probes:** clinic visits, employment, travel time, integration of treatment into routine

5. We understand that you talk a lot about adherence during counselling sessions. How do you think the SAT has impacted on your adherence, if at all?

**Probes:** positive/negative impacts

6. Can you tell me what you like most about taking your treatment at home, as well as what you find most challenging about it?

7. Is there anyone who gives you support for taking your treatment at home? Please tell me about this person and what kind of support they give you.

**Probes:** differences in support between SAT and clinic, relationship with person, type of support

8. Is there anything that you would like us to do differently with the SAT programme?

**Probes:** information, level of support, patient inclusion criteria, number of visits, ease of contacting clinic

9. Taking your treatment at home or at the clinic is an individual choice. Can you tell me how you would prefer to take your treatment, and why?

Thank you for your participation. Do you have any questions you would like to ask us?

**In-depth interview guide for patients (isiXhosa):**

**Isikhokhelo esinzulu sodliwano-ndlebe sezigulane:**

**Ukuxatyiswa kwenkqubo elingwayo yokuzinika unyango ngokwakho (SAT)**

Enkosi ngokuvuma ukuthetha nam namhlanje, nangokunikezela ngemvume yakho echaziweyo. Ndifuna ukukubuza ngamava akho ekuthatheni inxaxheba kwinkqubo elingwayo i-SAT eKhayelitsha. Zonke iimpendulo zakho ziya kugcinwa ziyimfihlelo yaye akunyanzelakanga ukuba uphendule nayiphi na imibuzo ongafuniyo ukuyiphendula. Akukho zimpendulo zichanekileyo nezingachanekanga kwimibuzo yam. Nceda uzive ukhululekile ukuba ungabuza imibuzo nangaliphi na ithuba leengxoxo.

1. Ungandicacisela ukuba wazi ntoni ngenkqubo elingwayo i-SAT obusoloko uthatha inxaxheba kuyo?

**Uphanda:** ukuqonda ngenkqubo, izizathu zenkqubo, iimfuno zokubandakanywa kwezigulane, ukubandakanyeka kwakho

2. Waziva njani xa wawubuzwa ukuba ungathanda na ukugoduka namayeza akho e-RR-TB?

**Uphanda:** ukuva kamnandi okanye kakubi, iinkxalabo nemibuzo, ukuqonda isizathu sokulungela

3. Abe njani amava akho okutyela amayeza akho ekhaya?

**Uphanda:** ubulula/ubunzima, ukutshintsha ukwenza, ukwazisa abanye, ubude bexesha lamayeza, iinkxalabo

4. Ungandicacisela ukuba indlela owenza ngayo izinto imihla ngemihla itshintshile oko waqala ukutyela amayeza akho ekhaya? Ungandicacisela ukuba iintsuku zakho zazinjani phambi kokuba kwenkqubo elingwayo, nangexesha layo?

**Uphanda:** utyelelo ekliniki, impangelo, ixesha lokuhamba, ukudityaniswa konyango nenkqubo yemihla ngemihla

5. Siyaqonda ukuba nithetha kakhulu ngokungayeki ukutya amayeza kwiiseshoni zokucebisa. Ucinga ukuba i-SAT ibe nafuthe lini ekungayekini kwakho amayeza, ukuba ibe nalo?

**Uphanda:** ifuthe elihle/elibi

6. Ungandixelela eyona nto uyithanda kakhulu ngokutyela amayeza akho ekhaya, kunye nento oyifumana ingumngeni kakhulu kuko?

7. Ukhona umntu okunika inkxaso ekutyeni amayeza akho ekhaya? Nceda undixelele ngalo mntu nohlobo lwenkxaso akunika yona.

**Uphanda:** umahluko phakathi kwenkxaso ye-SAT nkliniki, ubudlelane nomntu, uhlobo lwenkxaso

8. Ingaba ikhona into ofuna ukuba siyenze ngokwahlukileyo ngenkqubo ye-SAT?

**Uphanda:** ulwazi, iqondo lenkxaso, imfuno yokungeniswa kwesigulane, amatyeli otyelelo, ubulula bokudibana nekliniki

9. Ukutyela amayeza akho ekhaya okanye ekliniki yinto ozikhethele buqu. Ungandixelela ukuba ukhetha ukuwatya njani amayeza akho, nokuba kutheni?

Enkosi ngokuthatha kwakho inxaxheba. Ingaba unayo imibuzo ofuna ukusibuza yona?
